# Intrusion of episcleral buckles: report of two cases and brief review

**DOI:** 10.1186/s40942-020-00210-7

**Published:** 2020-04-09

**Authors:** Mohammad Zarei, Alireza Mahmoudi, Abdollah Hadi, Hamid Riazi-Esfahani

**Affiliations:** Retina service, Farabi Eye Hospital, Tehran University of Medical Sciences, Qazvin Square, South Karegar Street, Tehran, 1336616351 Iran

**Keywords:** Scleral buckling, Complication, Intrusion, Erosion, Vitreous haemorrhage

## Abstract

**Background:**

The authors report two cases of the scleral buckles intrusion and erosion that presented many years after primary surgery with vitreous haemorrhage in one of them. Although the erosion/intrusion of a silicone scleral buckle (SB) is rare, it may have serious consequences and optimal management can be challenging. Therefore, this diagnosis should be considered if attributable signs and symptoms including vitreous haemorrhage occurred after scleral buckling. The authors briefly review the literature on clinical presentation and management of the episcleral silicone buckling erosion and intrusion.

**Case presentation:**

Case 1: A 48-year-old woman with a history of scleral buckling for an inferior rhegmatogenous retinal detachment presented with visual loss in her right eye. A vitreous haemorrhage was observed. After Close observation, Partial resolution of haemorrhage revealed an intruded sponge segment in inferior vitreous cavity. Case 2: A 26-year-old man was referred for retinal evaluation. Twenty years earlier, he had undergone lensectomy for bilateral childhood cataract. Ten years ago, he had developed an aphakic RRD in the left eye. The detachment was managed with pars plana deep vitrectomy, endolaser, an encircling silicone band, and silicone oil injection. On examination an eroded band was noted.

**Conclusion:**

Although the erosion/intrusion of a silicone episcleral buckle is rare, it may have serious consequences and optimal management can be challenging. Unnecessarily destructive techniques may predispose the eye to this complication and should be avoided. Patients who have a history of SB need lifelong follow-up and this diagnosis should be considered if attributable signs and symptoms occurred.

## Background

Extrusion, erosion, and intrusion of buckling elements are rare but important complications of scleral buckling (SB) [1, 2]. Extrusion is the penetration of buckling element through Tenon’s capsule and conjunctiva externally. Erosion occurs when the element migrates internally and rests in subretinal space. Protrusion of the element into the vitreous cavity is called intrusion [2–4]. Suggested predisposing factors for intrusion includes myopia, glaucoma, thin sclera, multiple operations and infection [1, 3]. Scleral buckling erosion/intrusion may lead to retinal redetachment, pigment dispersion, vitreous haemorrhage, epithelial ingrowth, and endophthalmitis [4]. Here, we report two cases of erosion/intrusion of scleral buckles and review the literature.

## Case presentation

### Case 1

A 48-year-old woman presented with visual loss in her right eye. She reported similar episodes of transient visual obscuration in the same eye in recent 2 years with spontaneous resolution. Best corrected visual acuity (BCVA) was 20/200 in the right eye and 20/20 in the left eye. In the right eye, a vitreous haemorrhage blocking the fundus view was observed. Echography revealed an attached retina. The same eye had undergone cataract surgery and SB surgery, three and 29 years earlier, respectively. According to her surgical records, SB had been performed for an inferior rhegmatogenous retinal detachment (RRD): “after localizing, cryopexy was applied and a sponge segment was placed on the hole. Tire and band were passed beneath the muscles and fixed to the sclera by Mersilene sutures. Drainage of subretinal fluid was done via a 3 mm long sclerotomy which subsequently was closed with a mattress suture and treated with cryopexy”.

**Fig. 1:**
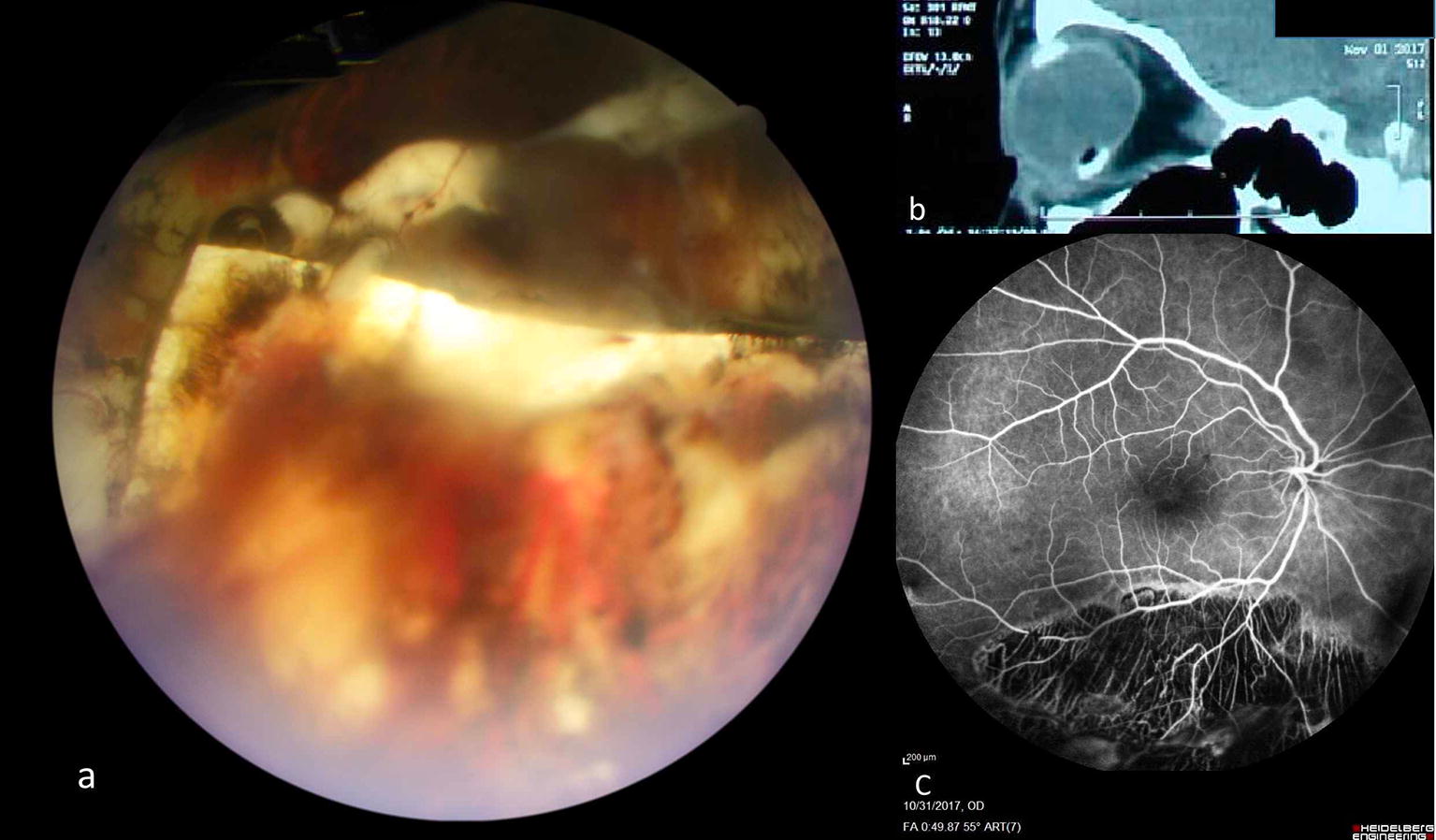
Case 1: **a** An intruded silicone sponge, partially covered with blood is seen in inferior vitreous cavity. Adjacent retina and choroid show extensive pigmentary and atrophic changes. **b** In sagittal orbital computed tomography (CT) scan, intruded hypodense silicone sponge is seen over the hyper dense silicone rubber (tire and band complex) in inferior globe. **c** Fluorescein angiography shows an extensive hypofluorescent area caused by severe chorioretinal atrophy in inferior retina

Close observation was scheduled. Partial resolution of haemorrhage revealed an intruded sponge segment in inferior vitreous cavity (Fig. 1a). To further clarify the pathology an orbital computed tomography was done (Fig. 1b). Fluorescein angiography was performed to rule out other causes of vitreous haemorrhage (Fig. 1c). To stabilize the retina as much as possible in case that buckle removal was needed, additional barrier laser was done. In following 16 months, despite multiple telephone recalls, patient did not comeback for followup visits. However, she reported good vision and no recurrence of symptoms.

### Case 2

**Fig. 2:**
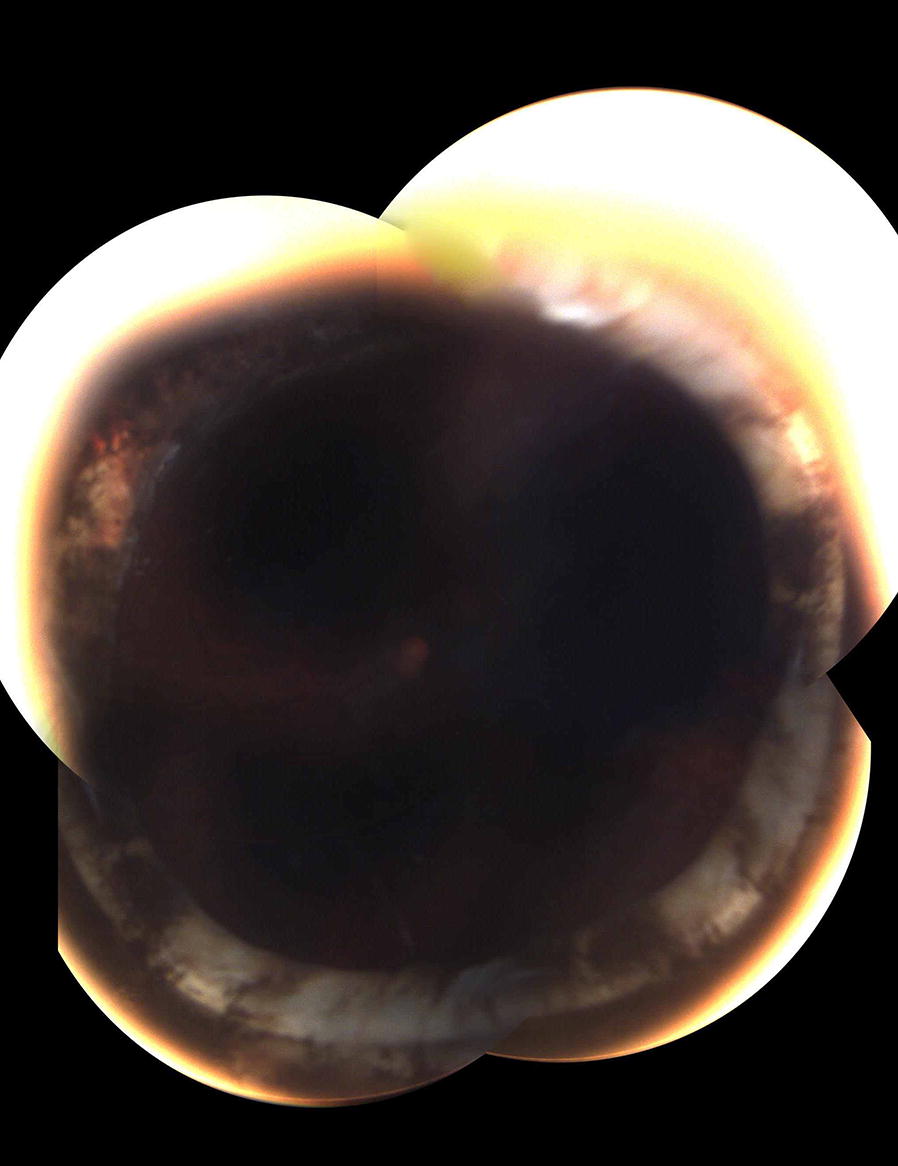
Case 2: Montage fundus photograph of the left eye. The band is seen to erode internally especially in temporal and inferior quadrants

An asymptomatic 26-year-old man was referred for retinal evaluation. Twenty years earlier, he had undergone lensectomy for bilateral childhood cataract. Ten years later he had developed an aphakic RRD in the left eye. The detachment was managed with placement of an encircling silicone band (band 240) 10–13 mm posterior to limbus, same session three port 20-gauge pars plana deep vitrectomy, 360° endolaser, and silicone oil injection. Three months later, silicone oil was removed following additional external laser photocoagulation. On examination, left eye had a BCVA of 20/28 and retina was attached. An eroded band was noted (Fig. 2). To address the possibility of progressive intrusion, 360° laser was applied posterior to the band. A segment of the band was then cut out surgically via an ab externo approach in supranasal quadrant. In 14 months of follow-up, retina remained attached and no changes in funduscopic appearance was noted.

## Discussion and conclusions

In early decades following introduction of SB surgery, erosion or intrusion of buckling elements into the eye were much more common. This complication was common when polyethylene tubes, Arruga sutures, and intrascleral silicone implant were used for scleral buckling which nowadays all can be considered obsolete [1]. The incidence of erosion/intrusion of buckling elements has decreased substantially since episcleral silicone sponges and rubber elements became the most common materials for the SB [5].

Here we reported two cases of erosion/intrusion of episcleral silicone buckling elements.

To better delineate predisposing factors, manifestations, and management, we reviewed reported cases of erosion/intrusion of “episcleral silicone buckling elements” in the literature [1, 3, 6–13] (Table 1). It is noteworthy that none of our cases have evidence of intrusion of anchoring sutures used in SB, therefore we are not discussing this type of complication which has been previously covered comprehensively elsewhere.

**Table 1:** Summary of reported cases with erosion/intrusion of episcleral buckles

Cases	Age (at buckling time)	Details of scleral buckling procedure	Interval time (years)^a^	Intruded element	Presentation	Visual acuity^b^	Management	Follow up duration (months)	Outcome
1. Nguyen et al. [1]	47	Encircling band (metal clips was used to hold the ends of band) + segmental sponge	10	Band and metal clip	Decreased VA	20/400	Observation	16	Progressed (the band was removal)
2. Birgul et al. [9]	77	Encircling band + cryopexy	4	Encircling band	Visual field defect	20/50	Observation	Not specified (loss)	Progressed
3. Ünlü et al. [3]	40	Encircling band + segmental radial sponge +cryopexy at horseshoe tear + drainage leading to incarceration of retina in drainage site; treated with additional cryopexy, radial sponge, pars plana vitrectomy and silicone oil tamponade	0.25	Segmental sponge	Incidental finding during routine follow-up	Not specified	Cutting of encircling band	36	Stable
4. Shetty et al. [12]	17	Encircling band +segmental tire	14	Encircling band	Vitreous haemorrhage (from neovascularization over the buckle)	20/40	Buckle removal +cutting of band^c^	3	Stable
5. Deramo et al. [6]	33	Encircling band + segmental sponge + Pars plana deep vitrectomy	1.5	Segmental sponge	Decreased VA + hypotony	4/200	Removal	8	Stable
6. Gray Arambura et al. [13]	65	First surgery: Segmental sponge + diathermy Second surgery: adding encircling buckle + diathermy	19	Encircling buckle	Vitreous haemorrhage	Not specified	Vitrectomy	6	Stable
7. Liang et al. [11]	37	Segmental sponge + cryopexy + drainage	3	Segmental sponge	Metamorphopsia and recurrent vitreous haemorrhage	20/20	Observation	6	NA
8. Shami et al. [8]	63	Encircling sponge	14	Encircling sponge (intruded at two sites)	Decreased VA	20/200	Observation	9	Stable
9. Gu et al. [10]	62	Encircling band + drainage + Dacron suture used to close the drainage site	15	Encircling band and Dacron knot	Decreased VA, vitreous haemorrhage, hyphema, and elevated IOP (in an aphakic myopic patient)	Light perception	Removal of Dacron knot and observation of intruded band	Not specified	Not specified
10. Mitra et al. [7]	Not specified	Pars plana deep vitrectomy combined with encircling band, followed by revisional vitrectomy for retina redetachment	Not specified	Encircling band	Incidental finding	6/24	Observation	Not specified	Not specified
11. Zarei et al. (current study)	19	Encircling band and tire with a segmental sponge + drainage + cryopexy + suturing and cryopexy of drainage site	29	Segmental sponge	Recurrent vitreous haemorrhage	20/200	Barrier laser	16	Stable
12. Zarei et al. (current study)	16	Pars plana deep vitrectomy combined with encircling band + endolaser photocoagulation + postoperative external laser photocoagulation	10	Encircling band	Incidental finding	20/28	Barrier laser + segmental removal of band	14	Stable

^a^The time interval between buckling surgery and buckle erosion/intrusion

^b^At time of erosion or intrusion

^c^Primary indication of surgery was anterior migration of buckle, not the intrusion

The mean interval time (from SB to erosion/intrusion) in reported cases is 10.88 ± 8.61 years (range = 0.25–29 years). This wide range may reflect variations in scleral thickness and resistance and variations in surgical details of SB procedures (e.g. cryopexy, diathermy, drainage of subretinal fluid and degree of tightening of the encircling elements) [14]. However, majority of cases are discovered more than 10 years after SB [5].

Eleven out of twelve patients were treated with a cerclage (with or without segmental elements): ten had an encircling band and one had an encircling sponge. It seems that prolonged circumferential inward force from a tight cerclage is a major contributing factor.

Six patients had an encircling band combined with a segmental sponge (four cases) or a tire (two cases). A rubber tire is wider than a rubber band. Therefore, compared to a tightened band alone, force exerted by a tightened band on a tire is distributed over a larger area of sclera. This mechanism leads to decreased pressure over the underlying sclera and may be protective against erosion/intrusion [6]. Interestingly, in one of two reported cases with encircling band and a segmental tire, the intruded element has been the band in the “opposite quadrant” of the segmental tire [12]. Second reported case of combined encircling band and tire is case 1 of our report, who had reminiscences of excessive surgical interventions: placing a segmental sponge over the break after cryopexy, cryopexy and suturing of the drainage site, and using encircling tire and band over the sponge. Widespread atrophic changes in fluorescein angiography in this case (Fig. 1c) also suggests excessive cryopexy. This suggests that in cases of compromised scleral resistance- either as a preexisting condition or as a consequence of destructive surgical interventions-the tire may not be enough to protect the sclera from pressure of a tightened encircling band.

Using diathermy or cryopexy has been documented in five patients [9, 11, 13] (Table 1). As mentioned earlier, our first case showed evidence of excessive treatment including intense cryopexy which may weaken the sclera.

It has been suggested that if the drainage sclerotomy under the buckling element is to be closed with sutures, absorbable sutures are preferred to nonabsorbable sutures [10]. According to surgical records of our case 1, drainage sclerotomy had been closed with a mattress suture, however, the nature of this suture was not mentioned.

A history of multiple ocular operations may be considered an additional risk factor for buckle erosion in our case 2.

The optimal management of scleral buckle erosion/intrusion should be tailored to each case. Severity, extent, symptoms and course of erosion/intrusion should be considered. As shown in Table 1, five patients were planned to be under observation initially. However, progression of intrusion is reported in two of them eventually [1, 9].

In our first case the vitreous haemorrhage resolved gradually and after applying peripheral laser, patient reported no recurring symptoms in the next 16 months.

Cutting of the band with or without total or segmental removal of buckling elements has been suggested for the management [12]. However, the benefits of these methods remains unproven [8, 9, 12]. In some cases, observation is warranted. One concern is that removing the intruded buckling element may leave the globe open; if the removal is planned, surgeon should be prepared to manage any possible scleral defect.

Although the erosion/intrusion of a silicone episcleral buckle is rare, it may have serious consequences and optimal management can be challenging. Unnecessarily destructive techniques may predispose the eye to this complication and should be avoided. Patients who have a history of SB need lifelong follow-up and this diagnosis should be considered if attributable signs and symptoms occurred.

## Data Availability

Not applicable. This is a case report.

## Abbreviations

SB: Scleral buckling
RRD: Rhegmatogenous retinal detachment
